# MIL-161 Metal–Organic Framework for Efficient Au(III) Recovery from Secondary Resources: Performance, Mechanism, and DFT Calculations

**DOI:** 10.3390/molecules28145459

**Published:** 2023-07-17

**Authors:** Guangyuan Hu, Zhiwei Wang, Weiye Zhang, Hongxing He, Yi Zhang, Xiujun Deng, Weili Li

**Affiliations:** Department of Chemical Science and Technology, Kunming University, Kunming 650214, China; guangyuanh@icloud.com (G.H.);

**Keywords:** selectivity, e-wastes, metal–organic framework, gold, adsorption, DFT calculations

## Abstract

The recovery of precious metals from secondary resources is significant economically and environmentally. However, their separation is still challenging because they often occur in complex metal ion mixtures. The poor selectivity of adsorbents for gold in complicated solutions prevents further application of adsorption technology. In this study, a Zr-based MOF adsorbent, MIL-161, was synthesized using s-tetrazine dicarboxylic acid (H_2_STz) as an organic ligand. MIL-161 demonstrated a high adsorption capacity of up to 446.49 mg/g and outstanding selectivity for gold(III) in a simulated electronic waste solution as a result of the presence of sulfur- and nitrogen-containing groups. In addition, the MIL-161 adsorbents were characterized using Fourier transform infrared (FT-IR), field emission scanning electron microscopy (FESEM), thermogravimetric analysis (TG), Brunner–Emment–Teller (BET), and X-ray photoelectron spectroscopy (XPS). Additionally, the adsorption kinetics, isotherms, and thermodynamics of the MOF adsorbents were also thoroughly examined. More importantly, the experimental results and DFT calculations indicate that chelation and electrostatic interactions are the main adsorption mechanisms.

## 1. Introduction

Due to its outstanding physical and chemical qualities, gold is extremely important in high-tech industries, like electronics, communications, aerospace, chemical, and medicine [1,2,3]. For example, gold is widely used as circuits and electrical contact points because of its stability and reliability in extreme temperatures and environments [4]. However, due to the limited reserves and valuable attributes of gold, the current supply is insufficient to satisfy the rising demand. Meanwhile, the excessive use of precious metals in modern industries has greatly contributed to the generation of electronic waste (e-waste) and wastewater, such as in the electricity and electronics industries [5,6,7,8]. Therefore, the importance of recovering gold from secondary resources is highlighted [9,10,11].

According to recent studies, recovering gold from e-waste is more economical than mining it from ore [12]. So far, a variety of technologies, including membrane filtration, extraction, adsorption, solvent extraction, precipitation, and ion exchange, have been applied to large-scale gold ion recovery from aqueous solutions [13,14]. Out of them, adsorption is considered the most promising method because of its low cost, easy operation, low energy consumption, and good recyclability. The disclosed adsorbents, which include minerals, activated carbon, chitosan resin, and nanomaterials, show effective gold ion adsorption capabilities [11,15,16]. However, the existing adsorption technology’s poor selectivity for low concentrations of gold in complicated solutions restricts its potential for further application. Therefore, the key to recovering gold from secondary resources is the development of adsorbents with superior adsorption capability, robust stability, and high selectivity.

In recent decades, interest in metal–organic frameworks (MOFs), commonly referred to as coordination polymers, has increased [17]. They play a significant role in various industries, including gas separation, storage, and metal ion adsorption, thanks to their large surface area, numerous active sites, and changeable porosity [18]. In recent years, MOFs have been widely used in wastewater treatment, for example, UiO-66 showed adsorption capacities of 120.0 mg/g for Pd(II) [19]. Jamali et al. utilized a lanthanide metal–organic framework to adsorb Pb(II) and Cu(II) [20]. The nano-porous MOF-5 has an adsorption capacity of 290 mg/g for copper ions [21]. Those studies have shown that MOFs have good adsorption capacity and stability in the process of recovered precious metals. However, due to the fact that secondary resources are usually complex metal ion mixtures, we need highly selective metal ion adsorbents.

Many different gold ion adsorbents have been created in recent years. The changes in adsorbents are mainly controlled by the active donor atoms or functional groups of organic ligands. Adding donor atoms or functional groups with a strong affinity for gold is a successful way to change MOFs in order to enhance their capacity for chemical adsorption [22]. The hard and soft acid and base (HSAB) theory [23] can describe how gold ions and functional groups are bonded. The strong bonds that gold tends to form with soft Lewis bases are because it is a soft acid. Therefore, functionalizing the adsorbent with soft Lewis bases can significantly improve its selectivity. Sulfur and nitrogen functional groups have a high affinity for gold with D-π conjugation effects in water and organic solvents [22]. Pyrazine dicarboxylic acid is rich in sulfur and nitrogen; therefore, using pyrazine dicarboxylic acid to synthesize MOFs with metals has stronger selectivity for gold(III).

**Scheme 1:**
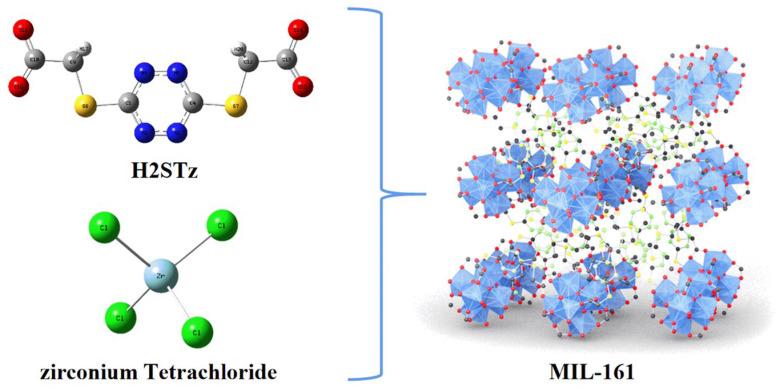
Preparation scheme of MIL-161.

Inspired by the above, we first synthesized a Zr-MOF with s-tetrazine dicarboxylic acid as an organic ligand for the recovery of gold from e-waste using a one-step solvothermal method, referring to the work of Paul Rouschmeyer et al. [24]. (Scheme 1). Additionally, by varying the pH level, adsorption period, and gold ion content, the adsorbent’s performance for adsorbing Au(III) was examined, as well as its thermodynamics, kinetics, isotherms, selectivity, and repeatability. Finally, XPS analysis and DFT calculations were used to examine the binding energy between Au(III) and MOFs in order to further understand the adsorption mechanism.

## 2. Results

### 2.1. NMR of H_2_STz

The H_2_STz organic ligand was characterized by nuclear magnetic resonance spectroscopy (NMR), and the NMR 1H (300 MHz, DMSO-d6): δ = 13.03 (s, 2H), 4.17 (s, 4H), 2.5 (DMSO), 0 (TMS) as shown in Figure S1. Nuclear magnetic resonance spectroscopy indicates that H_2_STz was successfully synthesized.

### 2.2. XRD and FT-IR of MIL-161

**Figure 1:**
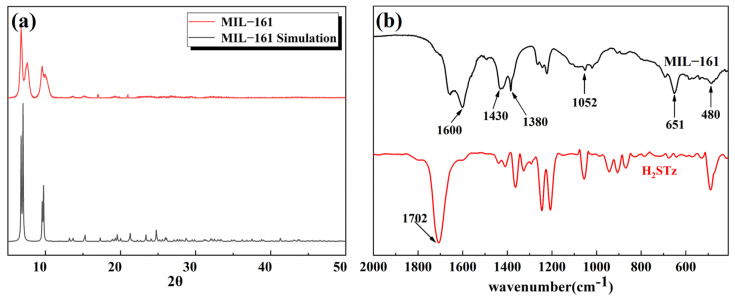
(**a**) PXRD patterns of MIL-161 and (**b**) FT-IR spectra of MIL-161 and H_2_STz.

The PXRD pattern of the synthesized MOF sample and the reference PXRD pattern of MIL-161 calculated from the literature data showed the same peak positions (Figure 1), as well as the experimentally obtained PXRD pattern of crystalline MIL-161 demonstrated in the supporting information (Figure S2). Also, the characteristic peaks of the prepared MIL-161 were in good agreement with the pattern reported by Paul et al. [24]. In summary, the PXRD results indicate the successful synthesis of MIL-161.

The IR spectrum of MIL-161 is shown in Figure 1b. The spectrum of MIL-161 (black) clearly showed that the peak at 1600 cm^−1^ was the binding peak of a carboxylic acid group and Zr^4+^ [25]. The Zr-N peak was at 651 cm^−1^, and the Zr-O vibrational peak was at 480 cm^−1^ [26,27]. The peaks at 1052 and 1380 were from the phenol, and the peaks at 1052 and 1380 were from the bending vibrational peak of the hydroxyl group and the C-O bond, respectively [28,29]. Furthermore, the presence of the ν (O-C-O) vibrational band centered at 1550 cm^−1^ and 1430 cm^−1^ was characteristic of the dicarboxylic acid group, while the characteristic band of the free carboxylic acid group without the H_2_STz ligand was at 1702 cm^−1^ (red line), which is consistent with previous studies [24]. Those results confirm the successful synthesis of MIL-161.

### 2.3. Morphology Characterization

**Figure 2:**
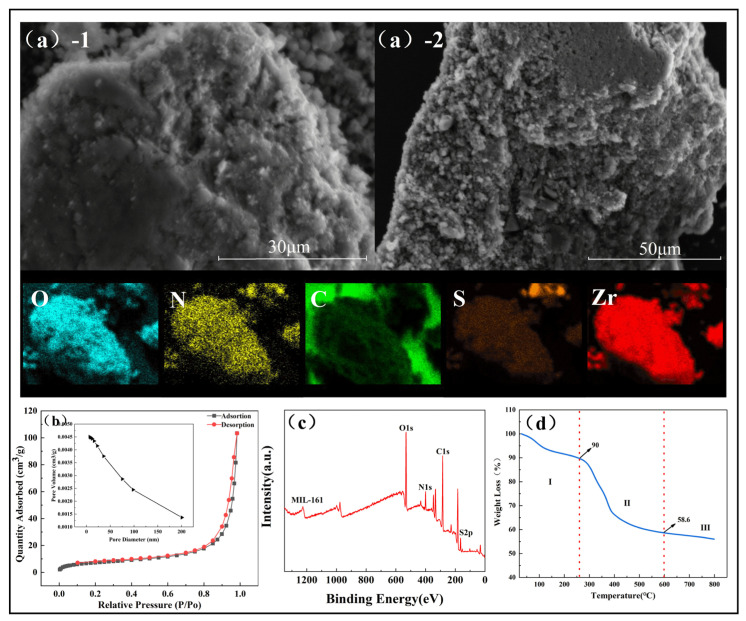
The TE-SEM (**a**) Figure (**a-1**) shows the 30 micron size and Figure (**a-2**) shows the 50 micron size, N2 adsorption isotherms (**b**), XPS (**c**), and TGA (**d**) of MIL-161.

Figure 2a displays MIL-161’s SEM. A rough polyhedral structure and irregular surface characterized the adsorbent’s surface. It is generally known that the adsorption capacity is determined by the pore-size distribution, which is assessed using N2 adsorption (a typical error is 10%) [30]. It establishes the quantity of ions in contact with the substance. As a result, the BET test has important practical implications for the adsorbent. An empty tube was filled with a 100 mg sample, and the adsorption and desorption isotherms of MIL-161 were upwardly convex in the low P/P0 region and rose rapidly in the higher P/P0 region, conforming to Type IV (Figure 2b). Table 1 demonstrates that MIL-161 has a specific surface area of 71.4192 m^2^/g. In addition, the average pore size of MIL-161 is 7.622 nm. The above proves that MIL-161 has many mesopores, providing many reaction sites for the adsorption of Au.

XPS and EDS are good tools for analyzing the surface element distribution. As shown in the total spectrum scanning of XPS (Figure 2c), the spectrum showed the peak of C1s, N1s, O1s, S2p, and Zr3d, indicating the presence of the above elements in MIL-161, and the EDS results provided a quantitative analysis of the above elements. The EDS results showed that the elements in MIL-161 were C (63.53%, wt%), N (3.59%, wt%), O (18.99%, wt%), S (1.4%, wt%), and Zr (15.49%, wt%). At the same time, we calculated the theoretical elemental content of MIL-161 and performed the conventional CHN(S) elemental analysis. As shown in Table 2, the weight percentages of C,N,H,S obtained from the CHN(S) elemental analysis were in general agreement with the calculated data. This outcome confirms the effective synthesis of MIL-161 and is compatible with the conclusion of the infrared spectroscopy. These results are consistent with the target components of the design and show that the adsorbent was successfully synthesized.

### 2.4. Thermal Stability Analysis

TG determined MIL-161’s thermal stability, and the thermal stability was examined between 25 and 800 °C at a heating rate of 10 °C/min in a nitrogen atmosphere. The thermogravimetric–differential scanning curve of MIL-161 is shown in Figure 2d. Three stages can be distinguished in MIL-161 (5 mg)-induced weight loss. Only 10% of the weight was lost during the first step (from room temperature to 253 °C), mostly as a result of physical water evaporation. In the second phase (253 °C to 600 °C), the weight loss rate reached 41.4%, mainly due to the combustion of organic matter. In this part, the structure of MIL-161 was destroyed. In the last stage, the weight loss can be ignored as the temperature increases, because when the leftover organic matter was burned, all that was left was Zr [11]. In summation, MIL-161 can maintain excellent thermal stability below 253 °C. From the perspective of the composition of organic ligands, MIL-161 may expose unsaturated sites, which provides a possibility for the adsorption of Au(III).

### 2.5. Adsorption Prformance

#### 2.5.1. Effect of pH

**Figure 3:**
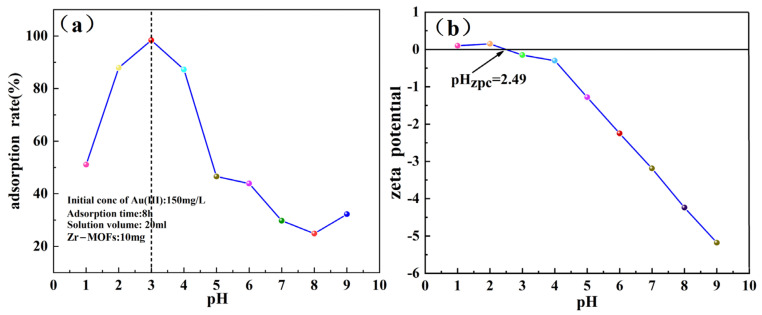
Impact of pH on adsorption (**a**) and Zeta potential (**b**) of MIL-161 (ion conc. of Au(III) = 150 mg/L; V/M = 10 mg/20 mL; T = 298 K; pH = 1–9; adsorption time = 8 h).

Because the pH of a solution influences both gold formation and the surface charge of the adsorbent, the pH of the solution has a significant impact on gold formation and adsorption [31], and in the pH range of 1.0 to 9.0, the effect of pH on Au(III) adsorption on MIL-161 was investigated. The results showed that the adsorption performance of MIL-161 varied significantly at different pH values. MIL-161 has good adsorption for Au(III) under acidic conditions, especially at pH = 3, where the adsorption rate was 98% (Figure 3a). In addition, the surface charge density of MIL-161 was investigated using the zero-point-charge (ZPC) technique in order to elucidate the interaction between the adsorbent and Au(III) in greater detail. When the pH was below pHzpc, the adsorbent surface was positively charged, and there was electrostatic repulsion between the adsorbent and the metal ion with the same charge. Conversely, when the pH was greater than pHzpc, the negatively charged adsorbent had an electrostatic attraction with cations, promoting the adsorption reaction [19]. As shown in Figure 3b, the pHzpc of the adsorbent was 2.49, which was less than the optimal pH. Clearly, at this pH, there is an electrostatic attraction between the adsorbent and the gold ions, which facilitates their capture.

#### 2.5.2. Adsorption Kinetics

**Figure 4:**
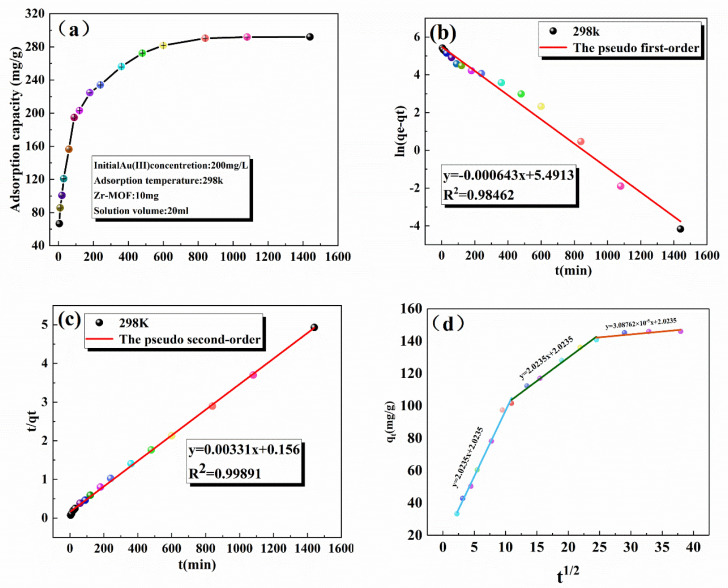
Effect of the reaction time on the adsorption Au(III) (**a**); pseudo-first-order model (**b**); pseudo-second-order model (**c**); Weber–Morris intra-particle diffusion rate (**d**).

Adsorption kinetics reflects the rate of the reaction and is utilized extensively to investigate adsorption mechanisms. To investigate adsorption kinetics, the adsorption equilibrium time of Au(III) on adsorbents at 25 °C, pH = 3.0, and an initial Au(III) concentration of 200 mg/L was measured. As illustrated in Figure 4a, before 1.5 h, with the extension of the adsorption time, the MIL-161 adsorption capacity rapidly increased. Between 1.5 h and 10 h, the adsorption rate of the adsorption capacity gradually slowed with the extension of time. After more than 10 h, the adsorption capacity tended to balance. This may be due to the fact that there are enough active sites interacting with the Au(III) at the beginning of the adsorption, leading to accelerated Au(III) absorption. As the active sites were progressively occupied, the adsorption capacity increased incrementally until saturation was achieved. To investigate the adsorption mechanism in greater depth and determine whether the interaction between MIL-161 and Au(III) was a result of physical or chemical adsorption, we employed the pseudo-first-order (PFO) (Equation (1)) and the pseudo-second-order (PSO) models (Equation (2)) [32,33] and the Weber–Morris model (Equation (3)) [34]. Three types of linear equations are represented as follows:

ln(q_e_ − q_t_) = lnq_e_ − k_1_t (1)

t/q_t_ = 1/(k_2_q_e_^2^) + t/q_e_ (2)

q_t_ = k_i_t^(1/2)^ + C (3)

where q_t_ (mg/g) and q_e_ (mg/g) are the adsorption capacities of Au(III) at time t (min) and at equilibrium, respectively; k_1_ (min^−1^) is the rate constant of the first-order model; k_2_ (g·min^−1^·mg^−1^) is the rate constant for the pseudo-second-order model at the equilibrium; k_i_ (mg·g^−1^ min^1/2^) is the rate constant for the Weber intra-particle diffusion model, respectively; and C gives the boundary layer thickness.

The linear relationship of the PFO and PSO models is shown in Figure 4b,c, respectively, and the resulting parameters are reported in Table 3. The results showed that the PSO model had a higher correlation coefficient (R^2^) than the PFO models, which can describe the kinetic behavior of the adsorption process. Also, the adsorption capacity obtained from the PSO model fitting was higher than that of the PFO model. These results showed that the data obtained from the adsorption process of Au(III) using the PSO model are well investigated. The parameter values of the PSO model of the adsorption of Au(III) on MIL-161 were determined to be 0.00331 mg/(g·min) and 302.114 mg/g, respectively, and this indicated that the prepared MIL-161 had a high adsorption.

The Weber–Morris model was developed to suit the MIL-161 adsorption amount versus the t^1/2^ curve. As shown in Figure 4d, the entire process can be separated into three linear regions: external surface rebinding, intragranular diffusion, and rebinding equilibrium. In the first stage, Au(III) swiftly diffused from the solution phase to the outer surface of MIL-161; the diffusion rate constant K1was 8.129 mg g^−1^min^0.5^. And in the second stage, Au(III) spreads further to the interior channel to adsorb in the mesopores of MIL-161; at this stage, K2 was 2.88 mg g^−1^min^0.5^, indicating a slower diffusion rate than in the first stage. In the final stage, the most active sites were occupied by Au(III) in order to reach the saturation state, and the rebinding equilibrium was achieved; the value of K3 at this stage was 0.359 mg g^−1^min^0.5^, and the diffusion rate was the slowest. According to the Weber–Morris model, the adsorption process of MIL-161 was complex and primarily determined by two rate-control phases [35].

#### 2.5.3. Adsorption Isotherms

**Figure 5:**
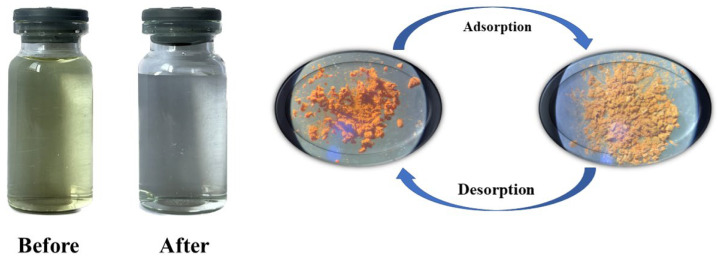
The pictures of the solution at 300 mg/L before and after adsorption by MIL-161; macro change in MIL-161 before and after adsorption.

**Figure 6:**
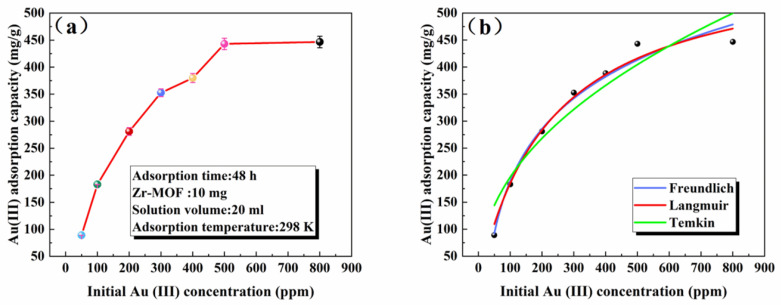
Effect of the initial Au(III) concentration on adsorption capacity (**a**) and the fitting curves of models (**b**).

Before and after adsorption, Figure 5 depicts the distinct alterations in the solution and prepared materials. Figure 6 depicts the Au(III) adsorption isotherms measured by MIL-161 at 25 °C, pH = 3.0, and initial Au(III) concentrations ranging from 50 to 800 mg/L. Figure 6a illustrates that the adsorption capacity of MIL-161 for Au(III) increased as the Au(III) concentration increased, with a maximal adsorption capacity of 446.49 mg/g. Table 4 displays the saturated sorption efficacy of MIL-161 and other Au(III) adsorbents. Clearly, MIL-161 demonstrated a greater adsorption capacity than other adsorbents.

The Langmuir isotherm model (Equation (4)), Freundlich isotherm model (Equation (5)), and Temkin model (Equation (6)) were used to study the adsorption isotherm data in order to study the adsorption mechanism of the adsorbent in greater detail [36,37]. The expressions for the three models are as follows:

Q_e_ = (Q_m_K_L_C_e_)/(1 + K_L_C_e_) (4)

Q_e_ = K_F_C_e_^(1⁄n)^ (5)

Q_e_ = (RTln(K_T_ C_e_))/β (6)

where Q_e_ (mg/g) is the adsorption capacity of Au(III) ion at equilibrium, C_e_ (mg/L) is the equilibrium concentrations of Au(III) in solution, Q_m_ (mg/g) is the maximal adsorption capacity of Au(III), and K_L_ (L/g) is a constant in the Langmuir model. K_F_ and n are the constants in the Freundlich model. In the Temkin model, K_T_ is the constant of the Temkin model; β, R, and T are the constant, temperature in Kelvin, and the constant of universal gas, respectively.

Figure 6b depicts the fitting curve, and Table 5 lists the fitting parameters of the model. The correlation coefficient of the Temkin model is 0.98, indicating that the interaction between the adsorbent and the adsorbed substance in various adsorption layers decreases the adsorption heat. The Langmuir model has a higher correlation coefficient (R^2^) than the Freundlich model (0.917%). This demonstrates that the adsorption isotherm is consistent with the Langmuir model and demonstrates single-layer adsorption behavior [22].

**Table 4:** The saturated adsorption capacity of different Au(III) adsorbents.

Adsorbent	pH Value	Adsorption Capacity (mg/g)	Literature
MIL-161	3	446.49	This work
MNP-G3	6.5	3.58	[38]
Thiol-ene hydrogels	0.5	118.8	[39]
APS-LCP	4	261.3	[14]
PS-APD resin	4	278.5	[40]
AIAC0.2M	1	191.92	[41]

#### 2.5.4. Thermodynamic Experiment

**Figure 7:**
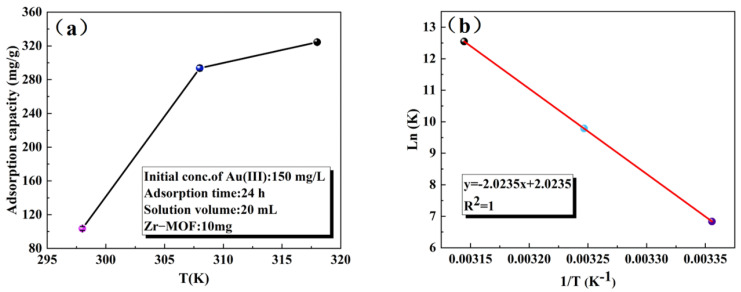
Effect of temperature on the adsorption (**a**) and plot of ln K_c_ versus 1/T (**b**).

Thermodynamic experiments were conducted at 298, 308, and 318 K to determine the thermodynamic behavior of the adsorption process (Figure 7a). The amount of Au(III) adsorbed on MIL-161 increased gradually along the temperature gradient (298–318 K), indicating that the increase in temperature could promote the adsorption. In order to further study the thermodynamic mechanism of the adsorption process, we introduced the Gibbs free energy equation (Equation (7)) and the van’t Hoff equation (Equation (8)) as follows [42]:

∆G = −RTln(1000∙K_c_) (7)

Q_e_ = K_F_C_e_^(1/n)^ (8)

where R is the universal gas constant (8.314 J mol^−1^ K^−1^), T is the absolute temperature (K), and K_c_ is the equilibrium constant (K_c_ = Q_e_/C_e_). The value of ΔG is calculated directly from the above equation, and the ΔH and ΔS values are obtained using the slope and intercept of ln K_c_ vs. 1/T, as shown in Figure 7b. The calculated values of the thermodynamic parameters are shown in Table 6.

As listed in Table 6, at various temperatures, H > 0 and G 0, and the positive value of H indicates that the adsorption of Au(III) on MIL-161 is an endothermic process. The temperature-induced change in the adsorption of Au(III) on the adsorbent was primarily owing to the physical adsorption of electrostatic forces. Therefore, it can be concluded that the rise in temperature accelerated the mass transfer of Au(III) and enhanced the adsorption behavior. At the same time, Gibbs decreased with increasing temperature, indicating that the increase in temperature favored the adsorption process [43]. In summary, the adsorption of Au(III) on MIL-161 was a spontaneous, endothermic, and entropy-increasing process.

### 2.6. Selectivity and Reusability

**Figure 8:**
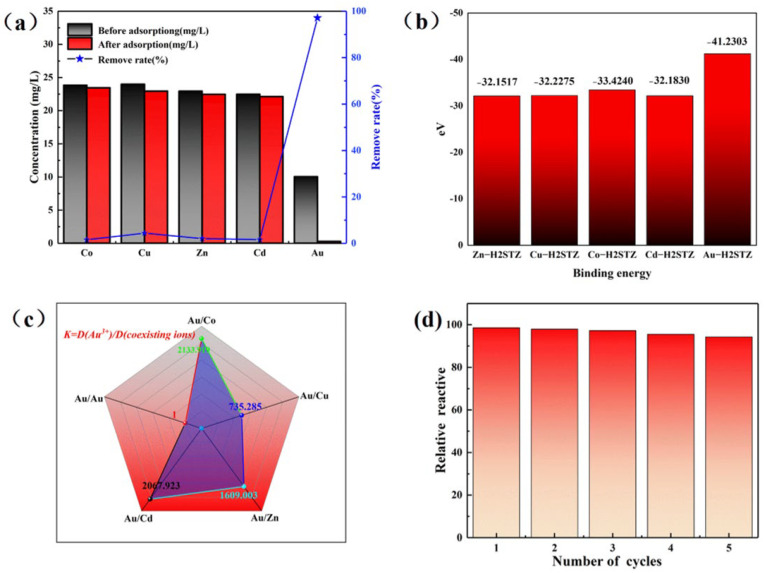
Selective (**a**); binding energy of Au(III) and other metals to H_2_STz (**b**); selectivity coefficients (**c**); reusability of MIL-161 for 98 mg/L Au(III) adsorption at 25 °C (**d**).

Various metal ions are frequently found in e-waste effluent. Consequently, it is essential to evaluate the removal efficacy of MIL-161 for Au(III) in the presence of multiple competing ions. The adsorption selectivity of MIL-161 on Au(III) was investigated by simulating e-waste leachate, and the effectiveness of the adsorbent was analyzed. Metal ion concentrations, including Zn(II), Cd(II), Au(III), Pb(II), and Cu(II), in the wastewater were measured before and after the adsorption experiments. As shown in Figure 8a, the removal rate of Au(III) was engaged to 97.1% in the environment where the concentration of other metal ions was twice higher than the concentration of Au(III), while the coexisting metal ions were all below 5%. These results indicate that MIL-161 is more likely to interact with Au(III) when adsorbing coexisting metal ions in wastewater. The selective adsorption of Au(III) by MIL-161 can be explained by the soft and hard acid–base (HSAB) theory, which states that adsorbents containing soft base functional groups (e.g., -S-) have good affinity for soft acids. Because there are many active sites containing S, N, and O in the organic ligand H_2_STz, and Au(III) belongs to one of the soft acids, MIL-161 can selectively adsorb large amounts of Au(III) [44].

To investigate this mechanism of high selectivity, the binding energies of Au(III) and other metals to H_2_STz were calculated using DFT. The binding energy of Au(III) to H_2_STz was greater than that of Co(II), Cu(II), Zn(II), and Cd(II), as shown in Figure 8b, indicating that the organic ligand H_2_STz which provides recognition sites had a coordination geometry selectivity for Au(III). The specific DFT calculation data are displayed in the supporting information.

To determine the adsorption selectivity of Au(III), the selective adsorption constant (D) and distribution coefficient (K) were employed to evaluate the metal ion selectivity. And D and K were calculated using the Equations (10) and (11) [45]. Figure 8a illustrates the outcomes of the selective experiment. MIL-161 demonstrated superior selective adsorption for Au(III) and the greatest removal rate. In Table 7 are depicted the calculated D and K values.

Au(III) has the highest D value (67,310.34 mL/mg), indicating that MIL-161 had the strongest affinity for Au(III). As well, the K values of MIL-161 for Au(III)/Co(II), Au(III)/Cu(II), Au(III)/Zn(II), and Au(III)/Cd(II) were 2133.92, 735.285, 1609.004, and 2067.923, respectively (Figure 8c). In conclusion, the results clearly showed that MIL-161 had a strong affinity and selectivity for Au(III), which means that MIL-161 was more likely to adsorb Au(III) in aqueous environments with multiple metal ions.

The reusability of adsorbents is a crucial factor in their practical applications. The results of the repeated adsorption experiments are shown in Figure 8d. After five cycles, the adsorption rate of MIL-161 to Au(III) only decreased by 4.3%. Moreover, PXRD, FTIR, and XPS were performed on the restored MIL-161 after adsorption of Au, the main characteristic peaks in the IR spectra were basically unchanged (Figure S3), and the Bragg peaks in PXRD mode were at the same positions as the original state (Figure S4), while the XPS total spectrum scan showed no change in the constituent elements of MIL-161 (5). This indicated that MIL-161 had good reusability performance, displaying a huge potential for gold recovery.

### 2.7. Adsorption Mechanism

**Figure 9:**
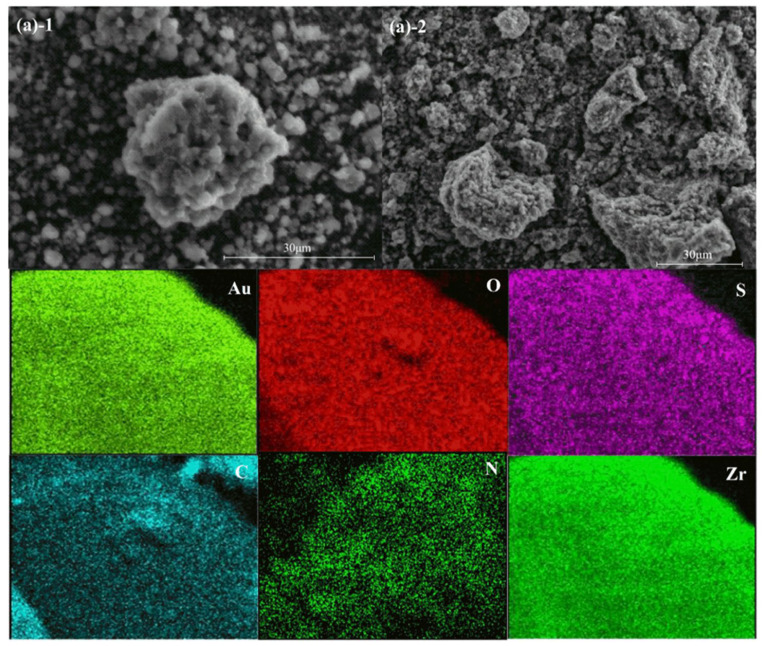
The TE-SEM Figure (**a-1**) shows the 30 micron size and Figure (**a-2**) shows the 50 micron size of MIL-161 after adsorption of Au(III).

**Figure 10:**
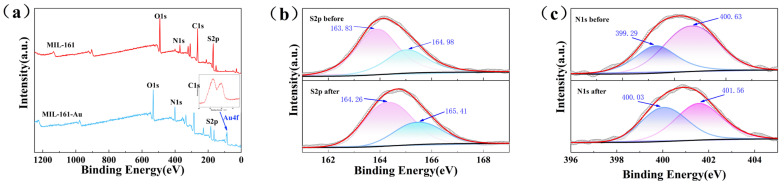
XPS spectra of MIL-161 before and after Au(III) adsorption (**a**), and high-resolution XPS of S2p (**b**) and N1s (**c**) of MIL-161 before and after adsorption of Au(III).

SEM-EDS was used to observe the microstructure of MIL-161 before and after adsorption to investigate the morphological changes. Compared with the image before adsorption of Au(III) (Figure 2a), the appearance of white particles after adsorption may be due to the adsorption of Au(III) (Figure 9a-1,a-2). In addition, Au(III) was detected in the EDS elemental analysis, and the EDS results showed that the elements in MIL-161 after adsorption of Au(III) were C (23.41%, wt%), N (9.1%, wt%), O (25.73%, wt%), S (9.67%, wt%), Zr (24.37%, wt%), and Au (7.72%, wt%). It was shown that the adsorbent successfully adsorbed Au(III). Figure 10 depicts the XPS analysis results of MIL-161 before and after Au(III) adsorption. Two peaks were observed for both N1s and O1s, and a new Au4f peak appeared in MIL-161-Au (Figure 10a). The presence of Au4f confirmed that Au(III) was successfully adsorbed onto MIL-161. Moreover, the binding energy of the S2p spectrum in MIL-161 can be separated into two peaks: S3p3/2 (163.83 eV) and S2p1/2 (164.98 eV) (Figure 10b). After Au(III) adsorption, the two peaks of S2p were simultaneously shifted to 164.26 eV and 165.41 eV; this indicates that Au(III) was coordinated to the S atom in the ligand H_2_STz. Similarly, Figure 10c illustrates that the binding energy of the N1s spectrum in MIL-161 can be divided into two peaks: C=N (400.63 eV) and C-N (399.29 eV). The C=N bond energy altered from 400.63 eV to 401.56 eV as Au(III) was adsorbed on MIL-161, while C-N shifted from 399.29 eV to 400.03 eV, which again suggested that Au(III) was coordinated to the N atom in the ligand H_2_STz [22]. In conclusion, the coordination of sulfur and nitrogen atoms of H_2_STz with Au(III) led to the adsorption of Au(III) on MIL-161.

**Figure 11:**
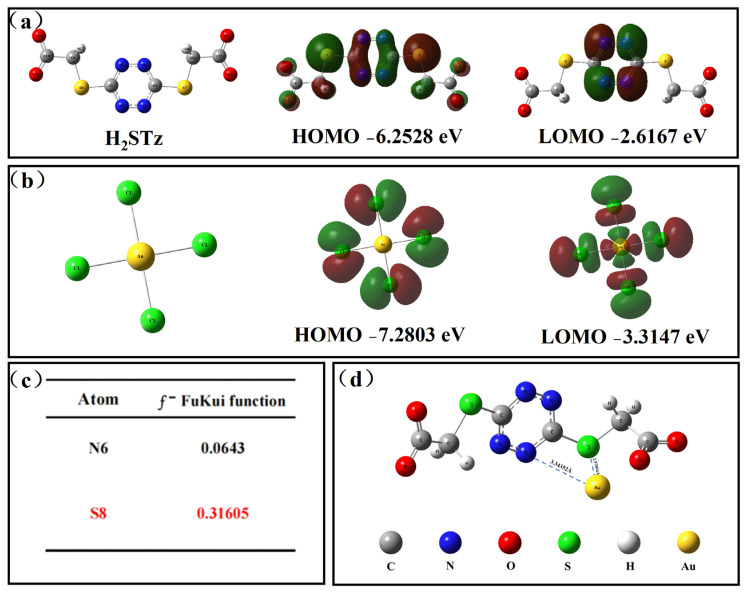
DFT calculations: H_2_STz geometry optimization, the HOMO energy, and the LOMO energy (**a**). Tetrachlorocuprate ion geometry optimization, energy of the HOMO, and energy of the LOMO (**b**), Fukui function (*f*^−^) (**c**), optimized geometry and bonding energy of H_2_STz (**d**).

The relationship between organic ligands H_2_STz and Au(III) in terms of binding properties was further revealed using HOMO-LOMO energy and Fukui function calculations. The geometrically optimized diagram of H_2_STz is shown in Figure 11a. The highest occupied molecular orbital of H_2_STz (HOMO, −6.2528 eV, Figure 11a) had an electron escape ability, and was easily attacked by AuCl_4_^−^, which formed coordination bonds with AuCl_4_^−^ at the lowest unoccupied molecular orbital (LOMO, −3.3147 eV, Figure 11b). In addition, the Fukui functions of H_2_STz are presented in Figure 11c. It can be seen that the S and N in H_2_STz had higher f-values, indicating that these atoms were more likely to provide electrons and be occupied by AuCl_4_^−^ to form adsorption sites, which was consistent with the results of the XPS analysis. Meanwhile, Au(III) had negative binding energy with N and S to form chemical bonds, indicating that gold ions were coordinated to the nitrogen and sulfur atoms of H_2_STz by chelating coordination, and the bond distances were 3.34352 Å and 2.57894 Å, respectively, as shown in Figure 11d. Changes in bond length and Wiberg bond order can also reflect the adsorption mechanism before and after Au(III) adsorption. After the adsorption of Au(III), the bond lengths of the C-S and C-N bonds adjacent to the N atom were lengthened and their Wiberg bond order was attenuated, as shown in Table 8. This occurred as a result of the transfer of electrons from S and N atoms to the empty orbitals of Au(III) following the adsorption of Au(III) [46].

## 3. Materials and Methods

### 3.1. Chemicals and Materials

Reagents used for synthesis and analysis, including deionized (DI) water (prepared in the laboratory), 1,4-benzene dithiol, zirconium(IV) chloride, 2-chloroacetic acid, N,N-dimethylformamide (DMF), were purchased from Aladdin Reagent (Shanghai) Co., Ltd., Shanghai, China. Tetrachloroauric acid (HAuCl_4_, 97%) was purchased from Shanghai Haohong Biotech Co., Ltd., Shanghai, China.

### 3.2. Instruments and Equipment

Using a pH meter (PHS-3C, Shanghai NESA, Shanghai, China), the pH value was determined. Fourier transform infrared (FTIR) spectra (Cary 6400, Agilent, Palo Alto, CA, USA) were used to conduct infrared measurements. The morphologies and microstructures of samples were examined using field emission scanning electron microscopy (FESEM; Quanta 400 FEG, FEI, Portland, OR, USA) and high-resolution transmission electron microscopy (HRTEM; JEOL JEM-2100, Hitachi, Tokyo, Japan). The analysis of thermal stability was performed using thermogravimetry (TG; 6300, Hitachi-chi, Tokyo, Japan). On an X-ray diffractometer (D2 PHASER, Bruker, Karlsruhe, Germany), X-ray diffraction (XRD) patterns were obtained. Utilizing a Thermo scalable 250Xi spectrometer, X-ray photoelectron spectroscopy (XPS) measurements were taken. Utilizing inductively coupled plasma atomic emission spectrometry (ICP-AES; Icap-6300, Thermo Scientific, Portland, OR, USA), the concentration of metal ions was determined.

### 3.3. Synthesis of Tetrazine Dicarboxylic Acid (H_2_STz)

The synthesis of H_2_STz was based on a reported procedure [47]. 3,6-Bis(3,5-dimethyl-1H-pyrazol-1-yl)-s-tetrazine (3.0 g, 11.1 mmol) was dispersed in 120 mL of acetonitrile. Added 1.65 mL (23.3 mmol, 2.1 eq.) of thioglycolic acid. The mixture was stirred at room temperature, changed from cloudy to crystalline and transparent (20 min), and formed an orange precipitate (1 h). The solid was filtered and rinsed with acetonitrile to yield an orange powder as the final product (1.21 g; 4.7 mmol; 40.3% yield).

### 3.4. Synthesis of MIL-161

**Scheme 2:**
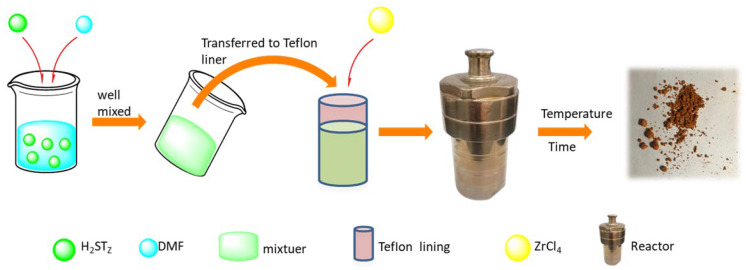
The steps involved in MIL-161 preparation.

Zirconium-based MOFs (MIL-161) were prepared using a typical hydrothermal method from a report [24]. A total of 197 mg (0.75 mmol, 1 eq.) of ZrCl_4_, 175 mg (0.75 mmol, 1 eq.) of H_2_STz, and 25 µL of 12 M hydrochloric acid were added to a reaction flask with Teflon lining. Then, 3.75 mL N,N’-dimethylformamide was added. After 14 h of heating at 80 °C, the mélange was cooled to room temperature. The solid obtained by centrifugation was washed four times with fresh DMF (4 × 10 mL) and once with 0.5 M hydrochloric acid (10 mL) and 30 h in an oven at 50 degrees Celsius. The process of preparation is depicted in Scheme 2.

### 3.5. Batch Adsorption Experiments

At 25 °C and 120 rpm, gold ion batch adsorption experiments were conducted. After each adsorption experiment, the sample was filtered through a 0.45 μm membrane, and its concentration was then determined. Using ICP-AES, the concentration of Au was determined. Each adsorption experiment was performed three times. The absorption capacity was computed using the following formula: Equation (9) [48]:

q = (C_0_ − C_e_)/1000W × V (9)

where q (mg/g) represents the adsorption capacity; C_0_ (mg/L) and C_e_ (mg/L) represent the initial and final concentrations of metal ions, respectively; V (mL) represents the volume of the solution; and W (g) represents the amount of sorbent.

In order to determine the adsorption kinetics of Au(III), 10 mg of MIL-161 was added to 20 mL of 200 mg/L Au(III) and stirred for 24 h. Then, certain amounts of the solution were taken out at different times (5 to 24 h). To calculate the Au(III) adsorption isotherm, 10 mg of MIL-161 was added to 20 mL of Au(III) solution with initial concentrations ranging from 50 to 800 mg/L and kept for 48 h. In the pH effect experiment, the adsorption performance under different pH values from 1 to 9 was investigated by adjusting the initial pH of the Au(III) solution by adding hydrochloric acid or sodium hydroxide.

To study the effect of interfering metal ions on MIL-161 adsorption of Au(III), 10 mg of adsorbent was mixed with the mixed solution containing Au(III) and evaluated the effect of coexisting cations, such as Zn(II), Cd(II), Au(III), Pb(II), Cu(II). Finally, using ICP-AES, the ion concentration in the filtrate was finally determined. The distribution ratio (D) and the selectivity coefficient (k) were computed using Equations (10) and (11), respectively [45]:

D = (C_0_ − C_e_)/C_e_ × V/W (10)

k = D_Au_/D_M_ (11)

where C_0_ (mg/L) and C_e_ (mg/L) are the initial and equilibrium metal ion concentrations, V (mL) is the volume of metal ion solution, W (g) is the mass of sorbent, and M represents other competitive metal ions.

### 3.6. DFT Calculation

The adsorption energy was determined by performing DFT calculations with Gaussian 16 W. The system was optimized in aqueous solution using a PBE1PBE/def2svp basis set and a D3(BJ) dispersion energy correction. Following convergence, the def2tzvp basis set was used to calculate the adsorption energy (E_ad_) [49]. The adsorption energy (E_ad_) between the H_2_STz molecule and different metal ions was calculated with Equation (12):

E_ad_ = E_(total)_ − E_(H2STz)_ − E_(adsorbates)_ (12)

where E_(total)_, E_(H2STz)_, and E_(adsorbates)_ are the total energy of the adsorption complex, the H_2_STz molecule, and the adsorbates, respectively.

### 3.7. Reusability Experiments

A total of 100 mg of MIL-161 was added to a 100 mL conical flask containing 20 mL of 100 mg/L Au(III) solution for the reproducibility experiment. The mélange was shaken at 25 °C for 24 h in an incubator with a constant shaking temperature. The supernatant was obtained by centrifugation, and the concentration of remaining Au(III) was determined. The remaining solid was mixed with 50 mL of a 10% pure thiourea solution and placed in a 25 °C incubator with constant stirring for 24 h. After centrifugation, it was rinsed with distilled water five times. The second experiment on adsorption was conducted. This procedure was performed five times.

## 4. Conclusions

In summary, we have synthesized Zr-based MOFs using s-tetrazine dicarboxylic acid as the organic ligand, which can be effectively applied to the recovery of Au(III) from complex solutions. Single-factor experiments were carried out on the effects of solution pH, reaction time, and initial Au(III) concentration. The maximum adsorption capacity of Au(III) was up to 446.49 mg/g at pH = 3. The wastewater experiments showed that MIL-161 had good selectivity for Au(III). After five adsorption–desorption cycles, MIL-161 showed good stability and repeatability, as well as high adsorption capacity and efficiency. The kinetics of Au(III) adsorption on MIL-161 are well described by the pseudo-second-order kinetics model, and the adsorption process followed the Langmuir isotherm model.

The characterization experiments and DFT calculations showed that the main adsorption mechanism of the adsorbent was electrostatic interaction and chelation, with the S and N of the organic ligands playing a major role in the adsorption process. These results suggest that MIL-161 has promising applications for gold recovery from wastewater.

## Figures and Tables

**Table 1:** Surface area of MIL-161.

Category	MIL-161
Surface area (m^2^/g)	71.4192
Average pore size (nm)	7.6221
Total pore volume (cm^3^/g)	0.00547

**Table 2:** Elemental analysis of MIL-161.

Calculated (wt%)	Experimental (wt%)
C 20.545%	C 21.390%
N 12.901%	N 15.790%
H 3.227%	H 2.004%
S 11.251%	S 17.176%

**Table 3:** The fitting parameters of kinetic models.

Kinetic Model	Parameters	Value
Pseudo-first order	q_e_	242.56
	k_1_	0.00643
	R^2^	0.98462
Pseudo-second order	q_e_	302.114
	k_2_	0.00331
	R^2^	0.99891

**Table 5:** The fitting parameters of isotherm models.

Isotherms Model	Parameters	Value
Langmuir	qm	602.1
	K_L_	0.004
	R^2^	0.983
Freundlich	K_F_	24.72
	1/n	0.448
	R^2^	0.917
Temkin	β	2.02
	K_T_	3.087
	R^2^	0.980

**Table 6:** Thermodynamic parameters for Au(III) adsorption.

Adsorbent	Temperature (K)	ΔG (kJ/mol)	ΔH (kJ/mol)	ΔS (kJ/mol·K)
MIL-161	298	−15.212	5.013	0.016
	308	−15.722		
	313	−16.233		

**Table 7:** D and K for coexisting ions.

Metal Ions	C_0_ − C_e_	D	k	Removal Rate (%)
Au(III)	9.76	67,310.344	-	97.1%
Co(II)	0.37	31.543	2133.92	1.6%
Cu(II)	1.05	91.543	735.285	4.4%
Zn(II)	0.47	41.833	1609.004	2.1%
Cd(II)	0.36	32.549	2067.923	1.6%

**Table 8:** Partial bond lengths and Wiberg bond order of MIL-161.

Items	Bond Lengths Å				Wiberg Bond Order			
	C4-S7	N5-N6	C1-N6	N2-N3	C4-S7	N5-N6	C1-N6	N2-N3
H_2_STz	1.74	1.31	1.333	1.288	1.549	1.789	1.776	1.917
H_2_STz-Au	1.751	1.301	1.34	1.295	1.409	1.862	1.678	1.882

## Data Availability

The data are contained within the article.

## Supplementary Materials

The following supporting information can be downloaded at: https://www.mdpi.com/article/10.3390/molecules28145459/s1, Figure S1. The ^1^H-NMR of H_2_STz. Figure S2. The PXRD pattern of the crystalline MIL-161. Figure S3. FT-IR spectra of MIL-161 restored to its original state after adsorption of Au. Figure S4. PXRD patterns of MIL-161 restored to its original state after adsorption of Au. Figure S5. XPS spectra of MIL-161 restored to its original state after adsorption of Au.
