# Supplementary material for: MIL-161 Metal–Organic Framework for Efficient Au(III) Recovery from Secondary Resources: Performance, Mechanism, and DFT Calculations
Source: Molecules. 2023 Jul 17;28(14):5459. doi: 10.3390/molecules28145459 (PMC10384270; doi:10.3390/molecules28145459)
Supplement: Supplementary file 1 [file molecules-28-05459-s001.zip › molecules-2430321-supplementary.pdf]

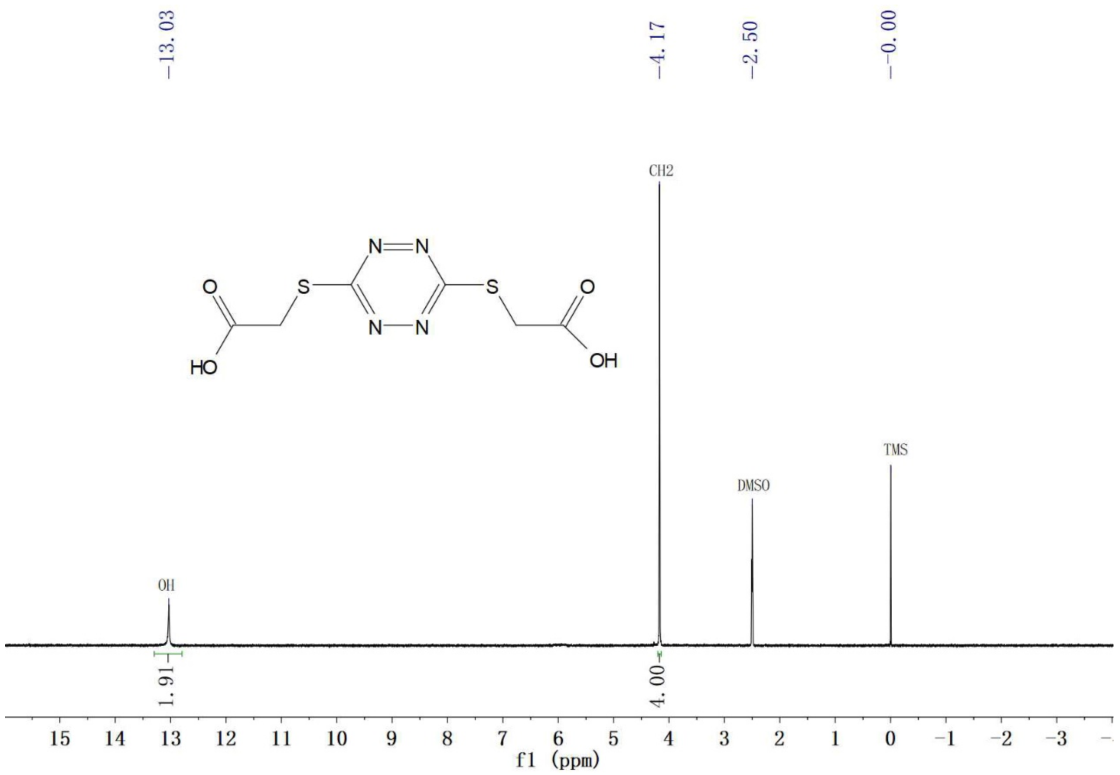

Figure S1. The ^{1}H-NMR of H_2STz.

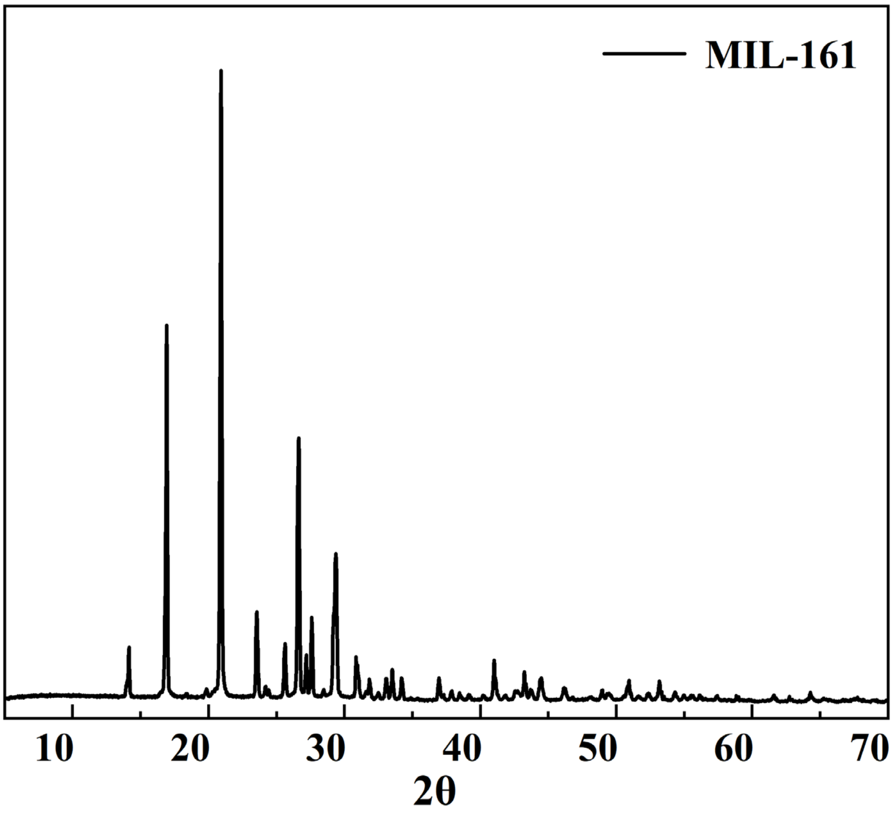

Figure S2. The PXRD pattern of the crystalline MIL-161.

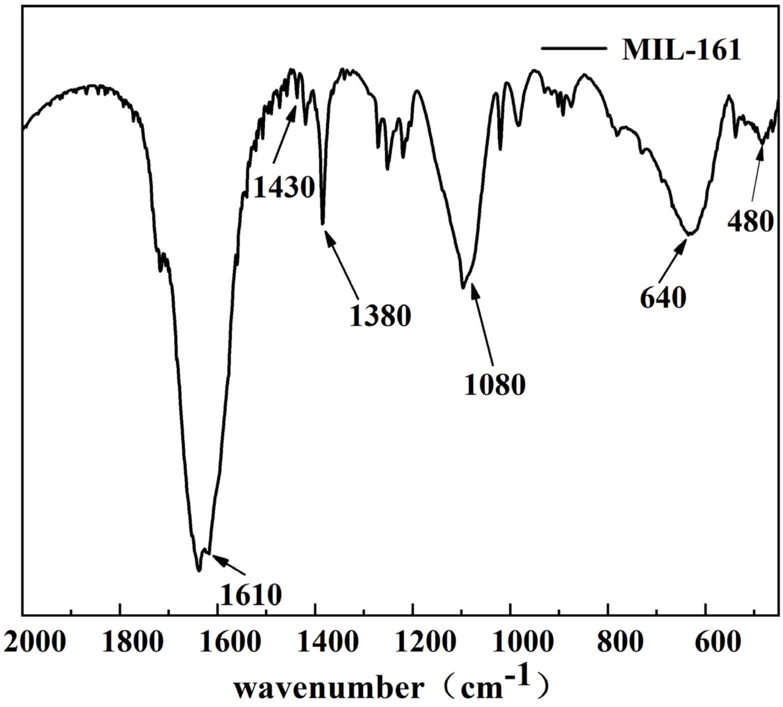

Figure S3. FT-IR spectra of MIL-161 restored to its original state after adsorption of Au.

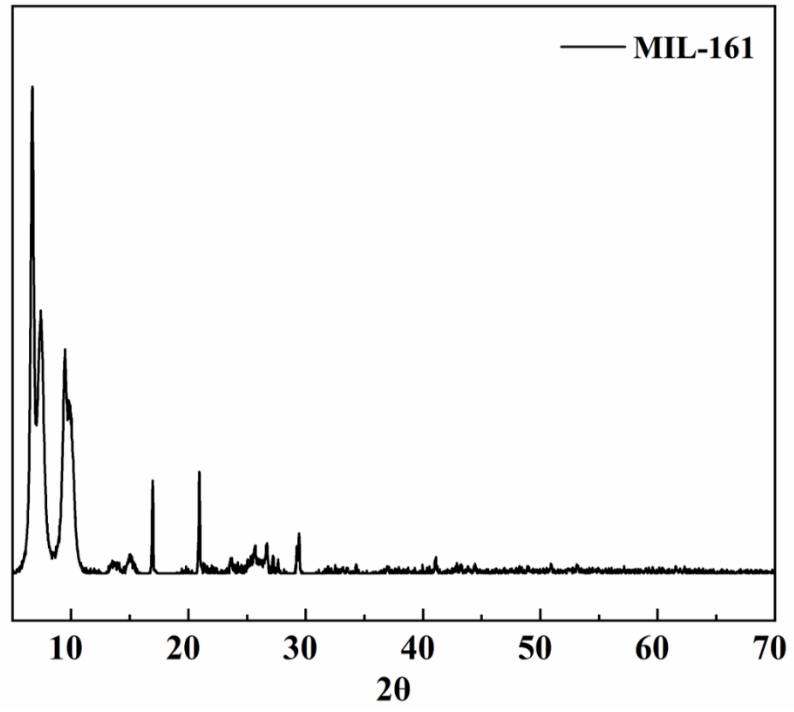

Figure S4. PXRD patterns of MIL-161restored to its original state after adsorption of Au.

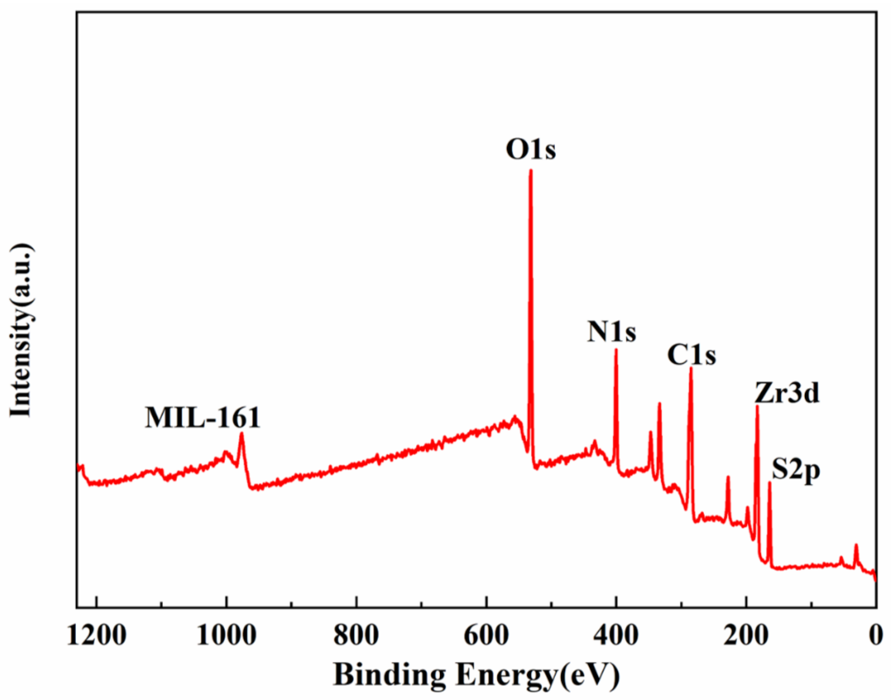

Figure S5. XPS spectra of MIL-161 restored to its original state after adsorption of Au.

Detailed data for DFT calculations:

Au：

Au	0.36942	0.46392	0.

Au-H_2STz：

C	-1	2.32529	1.1868	0.11298
N	-1	1.56073	2.14949	-0.4306
N	-1	0.31341	2.20145	-0.07773
C	-1	-0.13334	1.29282	0.79625
N	-1	0.677	0.50797	1.51149
N	-1	1.9311	0.45716	1.16792
S	-1	-1.83405	1.19339	1.19671
S	-1	3.92113	1.03091	-0.55169
C	-1	4.47235	-0.49565	0.24531
C	-1	5.86438	-0.87964	-0.28035
O	-1	6.30911	-1.9642	0.15409
C	-1	-2.61095	1.84326	-0.30988
C	-1	-3.51291	0.81926	-0.98041
O	-1	-4.71201	1.04852	-1.07609
O	-1	-3.0166	-0.29235	-1.43199
O	-1	6.42387	-0.08762	-1.0704
Au	0	-1.85399	-1.11711	0.05123
H	-1	4.51454	-0.36661	1.33651
H	-1	3.75065	-1.29833	0.02982
H	-1	-3.21921	2.71531	-0.04098
H	-1	-1.81413	2.16427	-0.99855

Cd-H_2STz

C	-1	1.75158	1.08322	-0.43465
N	-1	0.85234	1.34278	-1.39944
N	-1	-0.35443	1.64804	-1.03404
C	-1	-0.62866	1.67834	0.27475
N	-1	0.3161	1.66514	1.21892
N	-1	1.53039	1.36744	0.85801
S	-1	-2.26617	1.91981	0.84303
S	-1	3.28319	0.47534	-0.98
C	-1	4.04761	0.02164	0.59456
C	-1	5.41177	-0.63696	0.33603
O	-1	5.99475	-1.05663	1.35917
C	-1	-3.24186	1.25951	-0.53818
C	-1	-4.10595	0.08012	-0.12091
O	-1	-5.32471	0.17543	-0.19223
O	-1	-3.55504	-1.00964	0.32053
O	-1	5.81663	-0.68557	-0.84643
Cd	0	-1.59717	-1.75091	-0.02533
H	-1	4.18304	0.91088	1.22704
H	-1	3.38573	-0.67476	1.13171
H	-1	-3.89923	2.05353	-0.91254
H	-1	-2.54848	0.96409	-1.34079

Co-H_2STz

C	-1	-1.52757	-0.91467	-0.43417
N	-1	-0.61871	-1.13931	-1.3987
N	-1	0.59929	-1.39573	-1.03301
C	-1	0.87453	-1.41349	0.2758
N	-1	-0.06999	-1.43686	1.22001
N	-1	-1.2952	-1.18828	0.85882
S	-1	2.52038	-1.58876	0.84426
S	-1	-3.08223	-0.36904	-0.98015
C	-1	-3.86412	0.0557	0.59392
C	-1	-5.25347	0.65903	0.33469
O	-1	-5.85271	1.05634	1.35736
C	-1	3.46893	-0.89172	-0.53781
C	-1	4.28527	0.32171	-0.122
O	-1	5.50686	0.27502	-0.19329
O	-1	3.69134	1.38916	0.31816
O	-1	-5.65997	0.69003	-0.8478
Co	0	1.83589	2.05947	-0.04442
H	-1	-3.96394	-0.83749	1.22748
H	-1	-3.23054	0.77859	1.13019
H	-1	4.15744	-1.65933	-0.91125
H	-1	2.76429	-0.62517	-1.34073

Cu-H_2STz

C	-1	-1.58844	-0.88471	-0.47817
N	-1	-0.67406	-1.03997	-1.4511
N	-1	0.53774	-1.34061	-1.09871
C	-1	0.80159	-1.46983	0.20618
N	-1	-0.15115	-1.55959	1.13808
N	-1	-1.37028	-1.26705	0.78947
S	-1	2.4404	-1.71159	0.77058
S	-1	-3.13198	-0.27686	-0.98884
C	-1	-3.92229	0.02589	0.60903
C	-1	-5.30231	0.66552	0.39014
O	-1	-5.90561	0.98426	1.43763
C	-1	3.40869	-0.91437	-0.5419
C	-1	4.23544	0.25047	-0.02099
O	-1	5.45697	0.1949	-0.08669
O	-1	3.65015	1.28508	0.50149
O	-1	-5.69833	0.79909	-0.78877
Cu	0	1.97811	2.01976	-0.05942
H	-1	-4.03783	-0.91531	1.16576
H	-1	-3.28498	0.69419	1.20803
H	-1	4.09144	-1.65686	-0.97237
H	-1	2.71405	-0.57379	-1.3253

Zn-H_2STz

C	-1	-2.47859	1.43966	0.40764
N	-1	-1.6342	1.88269	1.35503
N	-1	-0.4857	2.34783	0.97084

C	-1	-0.2123	2.35081	-0.33847
N	-1	-1.13914	2.14513	-1.27809
N	-1	-2.29619	1.68608	-0.89876
S	-1	1.37269	2.80769	-0.92307
S	-1	-3.90327	0.63598	0.98831
C	-1	-4.58175	-0.01015	-0.55817
C	-1	-5.8317	-0.85351	-0.26172
O	-1	-6.33892	-1.40968	-1.25982
C	-1	2.42984	2.37614	0.48828
C	-1	3.46487	1.32119	0.13121
O	-1	4.65472	1.60377	0.19414
O	-1	3.08782	0.13891	-0.25088
O	-1	-6.23078	-0.90098	0.92275
Zn	0	1.22597	-0.67524	-0.01278
H	-1	-4.84701	0.81396	-1.23613
H	-1	-3.81914	-0.62529	-1.05979
H	-1	2.9573	3.27914	0.81863
H	-1	1.78499	2.02143	1.30717
